# The efficacy and safety of transcutaneous auricular vagus nerve stimulation for patients with minimally conscious state: a sham-controlled randomized double-blind clinical trial

**DOI:** 10.3389/fnins.2023.1323079

**Published:** 2023-12-14

**Authors:** Yifan Zhou, Yejing Sun, Pei He, Qi Xiong, Junwei Kang, Yunliang Tang, Zhen Feng, Xiaoyang Dong

**Affiliations:** ^1^Department of Rehabilitation Medicine, The First Affiliated Hospital of Nanchang University, Nanchang, Jiangxi, China; ^2^Rehabilitation Medicine Clinical Research Center of Jiangxi Province, Nanchang, Jiangxi, China

**Keywords:** transcutaneous auricular vagus nerve stimulation, minimally conscious state, electroencephalogram, somatosensory evoked potentials, brainstem auditory evoked potentials, P300 event-related potentials

## Abstract

**Background:**

Transcutaneous auricular vagus nerve stimulation (taVNS) has emerged as a potentially effective neuromodulation technique for addressing neurological disorders, including disorders of consciousness. Expanding upon our prior clinical study, which demonstrated the superior effectiveness of a 4-week taVNS treatment in patients with minimally conscious state (MCS) compared to those in a vegetative state/unresponsive wakefulness state, the aim of this investigation was to evaluate the safety and therapeutic efficacy of taVNS in individuals with MCS through a sham-controlled randomized double-blind clinical trial.

**Methods:**

A cohort of 50 adult patients (male = 33, female = 17) diagnosed with a MCS were randomly assigned to either the active taVNS (*N* = 25) or sham taVNS (*N* = 25) groups. The treatment period lasted for 4 weeks, followed by an 8-week follow-up period. The Coma Recovery Scale-Revised (CRS-R) and Glasgow Coma Scale (GCS) were administered at baseline and weekly during the initial 4 weeks. Additionally, the Disability Rating Scale (DRS) was used to assess the patients’ functional abilities via telephone at week 12. Furthermore, various neurophysiological measures, including electroencephalogram (EEG), upper-limb somatosensory evoked potentials (USEP), brainstem auditory evoked potentials (BAEP), and P300 event-related potentials (P300), were employed to monitor changes in brain activity and neural conduction pathways.

**Results:**

The scores for the active taVNS group in the CRS-R and GCS showed greater improvement over time compared to the sham taVNS group (CRS-R: 1-week, Z = −1.248, *p* = 0.212; 2-week, Z = −1.090, *p* = 0.276; 3-week, Z = −2.017, *p* = 0.044; 4-week, Z = −2.267, *p* = 0.023. GCS: 1-week, Z = −1.325, *p* = 0.185; 2-week, Z = −1.245, *p* = 0.213; 3-week, Z = −1.848, *p* = 0.065; 4-week, Z = −1.990, *p* = 0.047). Additionally, the EEG, USEP, BAEP, and P300 also demonstrated significant improvement in the active taVNS group compared to the sham taVNS group at week 4 (EEG, Z = −2.086, *p* = 0.037; USEP, Z = −2.014, *p* = 0.044; BAEP, Z = −2.298, *p* = 0.022; P300 amplitude, Z = −1.974, *p* = 0.049; P300 latency, t = 2.275, *p* = 0.027). Subgroup analysis revealed that patients with MCS derived greater benefits from receiving taVNS treatment earlier (CRS-R, Disease duration ≤ 1-month, mean difference = 8.50, 95% CI = [2.22, 14.78], *p* = 0.027; GCS, Disease duration ≤ 1-month, mean difference = 3.58, 95% CI = [0.14, 7.03], *p* = 0.044). By week 12, the active taVNS group exhibited lower Disability Rating Scale (DRS) scores compared to the sham taVNS group (Z = −2.105, *p* = 0.035), indicating a more favorable prognosis for MCS patients who underwent taVNS. Furthermore, no significant adverse events related to taVNS were observed during treatment.

**Conclusion:**

The findings of this study suggest that taVNS may serve as a potentially effective and safe intervention for facilitating the restoration of consciousness in individuals diagnosed with MCS. This therapeutic approach appears to enhance cerebral functioning and optimize neural conduction pathways.

**Clinical trial registration:**

http://www.chictr.org.cn, Identifier ChiCTR2200066629.

## 1 Introduction

Disorder of consciousness (DOCs) is a prevalent clinical complication characterized by prolonged periods of impaired awareness that ensue severe brain injuries or neurological impairments, including traumatic brain injury (TBI), stroke, hypoxic–ischemic encephalopathy (HIE), and related conditions (Li et al., 2016; Hernandez et al., 2021; Kowalski et al., 2021). These DOCs can be categorized into four distinct classifications based on their neurobehavioral function: coma, vegetative state/unresponsive wakefulness state (VS/UWS), minimally conscious state (MCS), and emergence from MCS (EMCS; Eapen et al., 2017). The VS/UWS condition is characterized by the manifestation of arousal, specifically eye opening, accompanied solely by reflexive behaviors (Monti et al., 2010). On the other hand, the MCS condition is distinguished by the presence of distinct and variable indications of awareness that can be perceptibly observed at the patient’s bedside, such as visual pursuit and the ability to follow commands (Thibaut et al., 2020). The implementation of advanced medical treatment and healthcare has significantly augmented the survival rate of patients following severe brain injury. It has been estimated that approximately 2.9% of patients with severe brain injury may develop to DOCs, resulting in an annual diagnosis of 300,000 individuals in the United States (Strauss et al., 2000). Currently, there is a lack of precise epidemiological data on patients with DOCs in China, with an estimated annual incidence of approximately 100,000 new cases (Zhao, 2018). Bedridden individuals with DOCs are susceptible to various complications, including pneumonia, pressure ulcers, and deep vein thrombosis, resulting in significant economic burdens and psychological distress for both families and society (Septien and Rubin, 2018; Proia-Lelouey and Boissel, 2023). Consequently, it is imperative to implement more proactive strategies for the rehabilitation of patients with DOCs.

The management of patients with DOCs presents significant considerations, particularly regarding the available therapeutic interventions. Presently, the therapeutic options for DOCs encompass pharmacological treatments, hyperbaric oxygen therapy, sensory stimulation, and neuromodulation techniques (Thibaut et al., 2019; Wu et al., 2021). While the majority of studies investigating interventions aimed at enhancing patients’ level of consciousness and functional recovery have primarily consisted of behavioral and brain imaging open-label trials and case reports, a number of randomized controlled trials have also been conducted, with a specific emphasis on the effects of pharmaceutical agents or the utilization of noninvasive brain stimulation (Thibaut et al., 2014; Wu et al., 2019; Fan et al., 2022). However, it is worth noting that only two studies have provided class II evidence regarding the efficacy of amantadine and transcranial direct current stimulation (Giacino et al., 2012; Thibaut et al., 2014). Despite the potential value of novel therapeutic approaches for individuals with DOCs, further investigation and validation are required to assess the effectiveness of optimized stimulation parameters, alternative pharmaceutical interventions, and rehabilitation strategies. This research is crucial for enhancing the rehabilitation process and ultimately enhancing the quality of life for these patients.

Research has increasingly focused on neuromodulatory therapies due to the limited effectiveness of available medicine for DOCs. The aforementioned interventions encompass non-invasive brain stimulations such as transcranial direct current stimulation, repeated transcranial magnetic stimulation, transcutaneous auricular vagal nerve stimulation (taVNS), and low intensity focused ultrasound pulse. Additionally, invasive brain stimulation methods, namely deep brain stimulation or vagal nerve stimulation (VNS), as well as sensory stimulation programs are also included (Xia et al., 2018). TaVNS, a novel form of VNS, has been extensively studied in both healthy individuals and people with neurological disorders, including those with drug-resistant epilepsy, migraine, and depression (Ellrich, 2019; Liu et al., 2020; Ridgewell et al., 2021; von Wrede and Surges, 2021; Wang et al., 2021a). It is considered a safe, non-invasive, and user-friendly therapeutic option, serving as an alternative to invasive VNS. Recent research has shown that both taVNS and VNS have the potential to attenuate brain damage and provide neuroprotection in rats with severe brain injury. This is achieved through the inhibition of oxidative stress, inflammation reaction, apoptosis, and reduction of blood–brain barrier permeability and brain edema (Tang et al., 2020; Wang et al., 2021; Bonaz, 2022; Divani et al., 2023). Our previous research has provided additional evidence supporting the neuroprotective effects of VNS in promoting the recovery of consciousness. Specifically, VNS has been found to activate the neuronal activity of the orexin-A and orexin 1 receptor in comatose rats induced by TBI (Dong et al., 2018; Dong and Feng, 2018). Moreover, our previous clinical investigations have demonstrated that a 4-week treatment of taVNS may be more effective in facilitating the recovery of consciousness in patients diagnosed with MCS than those with VS/UWS (Zhou et al., 2023). To date, studies on taVNS have yielded encouraging findings in individuals diagnosed with MCS. However, the absence of extensive randomized controlled clinical trials and the lack of clarity regarding the underlying mechanisms of consciousness restoration persist as significant gaps in current research (Noe et al., 2020; Osinska et al., 2022).

In light of these findings, we employed taVNS in a novel randomized, double-blind, sham-controlled trial to investigate its efficacy and safety in enhancing recovery from a MCS. Our hypothesis posited that a four-week course of taVNS therapy in MCS patients would lead to improvements in consciousness levels, while also being well-tolerated and devoid of significant complications.

## 2 Methods

### 2.1 Study design

**Figure 1:**
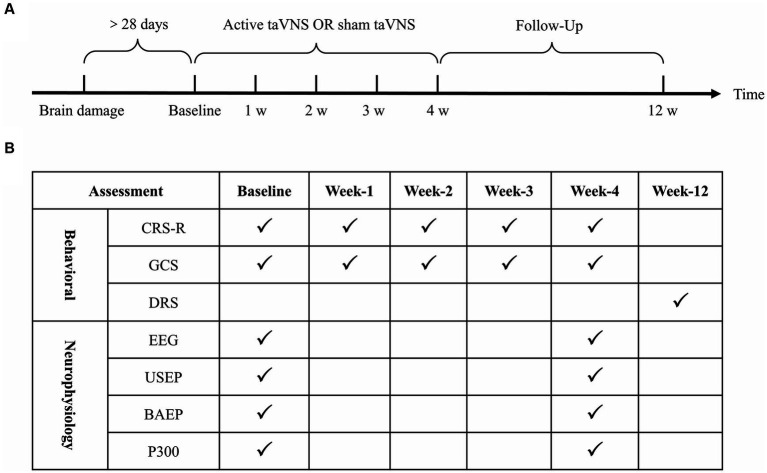
Clinical trial procedures and assessment time points. **(A)** Clinical trial procedures. **(B)** Assessment time points. taVNS, transcutaneous auricular vagus nerve stimulation; CRS-R, Coma Recovery Scale-Revised; GCS, Glasgow Coma Scale; DRS, Disability Rating Scale; EEG, electroencephalogram; USEP, upper-limb somatosensory evoked potentials; BAEP, brainstem auditory evoked potentials. P300, P300 event-related potentials.

In this study, a prospective, single-center, double-blind, sham-controlled, parallel-group design was employed. All patients were randomly assigned to either the active taVNS group (*n* = 30, conventional rehabilitation treatments + taVNS) or the sham taVNS group (n = 30, conventional rehabilitation treatments + sham taVNS) in a 1:1 ratio. Patients in the sham taVNS group underwent a taVNS procedure, but without any current output. Prior to enrollment, demographic information of all eligible patients were collected from the inpatient medical record system. Additionally, the level of consciousness and neuro-electrophysiological function of all patients were evaluated using the Coma Recovery Scale-Revised (CRS-R), Glasgow Coma Scale (GCS), upper-limb somatosensory evoked potentials (USEP) of bilateral upper limb, brainstem auditory evoked potentials (BAEP), P300 event-related potentials (P300) and electroencephalogram (EEG) before their inclusion in the study. All participants underwent a 4-week treatment period following enrollment, followed by an 8-week follow-up period. The primary measures of interest, namely the CRS-R and the GCS, were assessed at 1, 2, 3, and 4 weeks after enrollment. Additionally, EEG, USEP, BAEP and P300 were evaluated at the 4-week mark after treatment initiation. The Disability Rating Scale (DRS) was assessed during the 12th week of the follow-up phase (Figure 1).

The process of randomization was conducted by a doctor who was not involved in the trial, utilizing the group concealment method to ensure independence. The information pertaining to the assigned groups was kept undisclosed to all trial participants. The enrollment of patients with MCS was carried out by a rehabilitation physician, who subsequently assessed them using CRS-R and GCS scores, while ensuring that the patients remained unaware of their treatment allocation. The administration of taVNS was performed by specialized treatment technicians. Doctors in the examination room conducted EEG, USEP, BAEP, P300, and EEG tests without any knowledge of the treatment or grouping. An analysis of data is carried out by statisticians who do not know the allocation of treatments.

### 2.2 Subjects

This study was carried out at the Department of Rehabilitation Medicine, the First Affiliated Hospital of Nanchang University, between January 2023 and July 2023, with the inclusion of patients diagnosed with MCS. The criteria for identifying MCS patients encompassed the capacity to engage in visual tracking, execute directional voluntary movements, and perceive pain localization, while lacking the ability to effectively communicate with others in a functional manner (Giacino et al., 2002). This study adheres to the Consolidated Standards of Reporting Trials (CONSORT) guidelines. The inclusion and exclusion criteria are presented in Table 1. The demographic information of the patients was obtained from the inpatient electronic medical record system. Informed consent was obtained from the legal guardians of the patients. This clinical study, conducted at a single center, was registered with the Chinese Clinical Registry (ChiCTR2200066629) and approved by the Ethics Committee of the First Affiliated Hospital of Nanchang University ((2022)CDYFYYLK(06-031)) as well as conducted according to the Declaration of Helsinki.

**Table 1:** Study inclusion and exclusion criteria.

Inclusion criteria	Exclusion criteria
Age: >18 years old	Unstable vital signs
Duration of loss of consciousness >28 days	Severe pulmonary infection
Diagnosed with MCS as defined by the CRS-R	High intracranial pressure
A diagnosis of MCS based on the two CRS-R assessments performed during screening	History of previous neurological disorder prior to the brain injury
Acquired brain damage of known etiology	Deep sedation such as one caused by general anesthetics or a combination of central acting sedatives
Intact ear skin	Documented pregnancy
Informed consent given by the substitute decision maker	Active implant such as pacemaker or cochlear implant

CRS-R, Coma Recovery Scale-Revised; MCS, Minimally Conscious State.

### 2.3 Intervention

**Figure 2:**
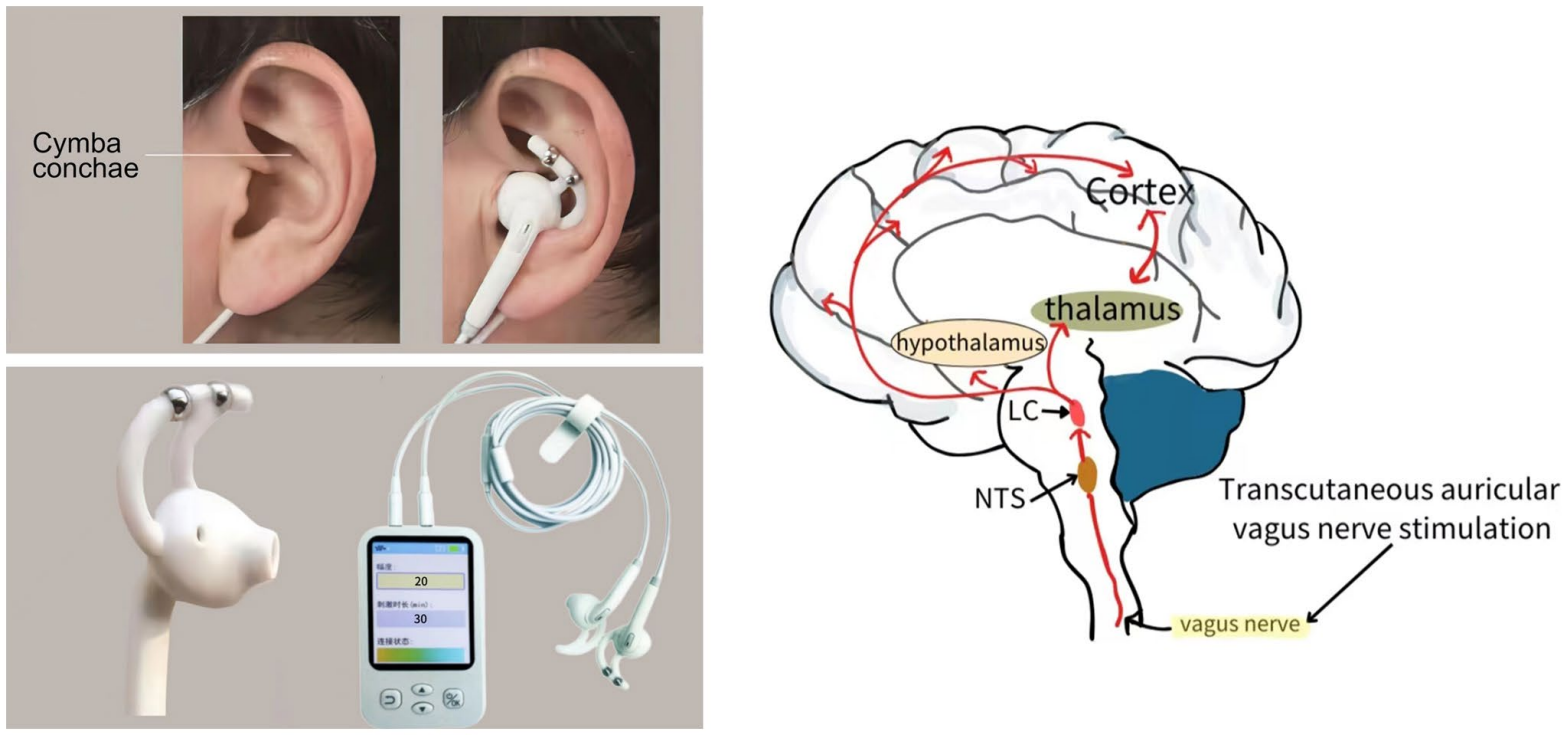
The schematic diagram of transcutaneous auricular vagus nerve stimulation implementation and potential conduction pathway.

Following the screening process, all patients were assigned to receive either active taVNS or sham taVNS. In the active taVNS group, specific auricular acupoints, including the cymba conchae within the vagus nerve distribution, were stimulated using an electrical stimulator on the left ear (taVNS501, Changzhou Rishena Medical Device Co., Ltd., Jiangsu, China; Figure 2). The parameters employed in this study were consistent with those utilized in our previous research, namely a sinusoidal waveform, pulse width of 200 microseconds, frequency of 20 Hz, and an intensity ranging from 15 to 20 gear. Initially, the intensity of taVNS was set at 20 gear, and subsequently adjusted using the nociception coma scale-revised (NCS-R) to determine the appropriate stimulation intensity. Pain presence was identified when the total score reached or exceeded 2, prompting a reduction of one level in order to ascertain the suitable stimulus intensity. The stimulation was administered for a duration of 30 min, twice daily, 6 days per week, over a period of 4 week (Zhou et al., 2023). In addition to receiving conventional treatments aligned with current guidelines, patients in the sham taVNS group also underwent the same procedure as the active taVNS group without current output, while being exposed to multimodal sensory and auditory stimulation, bedside conventional physical therapy, and environmental enrichment therapy (Giacino et al., 2018; Kondziella et al., 2020).

### 2.4 Sample size

The sample size for this study was determined using the Power Analysis and Sample Size (PASS) software (Version: 15.0), taking into account the difference in the rate of improvement in CRS-R scores observed in our previous clinical trial (Zhou et al., 2023). In the previous experiment, the sham-taVNS group demonstrated an improvement of 44.83%, while the active-taVNS group exhibited a higher improvement rate of 85.71%. To achieve a power of 90% in detecting a difference of 0.1 between the proportions of the two groups, a sample size of 23 participants per group was determined. The power analysis was conducted with a significance level (α) of 0.025 (one-sided), and an estimated loss to follow-up of 20%.

### 2.5 Assessment

This study employed the CRS-R and GCS scale to assess the behavioral function of patients with MCS on a weekly basis for the initial 4 weeks. The DRS was utilized for an 8-week follow-up at week 12, either through outpatient follow-up or telephone interviews. Furthermore, neuroelectrophysiology techniques, such as EEG, USEP, BAEP, and P300, were employed to identify alterations in brain function following treatment at baseline and week 4.

#### 2.5.1 Behavioral scale assessment: CRS-R, GCS, and DRS

The CRS-R scale is widely regarded as a highly effective assessment instrument for the diagnosis and ongoing monitoring of functional recovery of consciousness. It is considered the benchmark for differentiating between the status of VS/UWS and MCS (Giacino et al., 2004; Zhang et al., 2019). The scale comprises six subscales specifically designed to evaluate levels of arousal, auditory and verbal comprehension, expressive speech, visual perception, motor function, and communication. The total score on this scale is 23. The scoring system relies on a standardized approach aimed at eliciting the patient’s response and monitoring their behavioral reactions. Elevated scores are indicative of heightened levels of consciousness and neurological functioning. In order to enhance diagnostic precision, two proficient physicians conducted the assessment three times daily over the course of two consecutive days. The level of consciousness was determined based on the most favorable outcome among all evaluations.

The GCS is a well-established instrument utilized for the evaluation and quantification of a patient’s state of consciousness (Mehta et al., 2019). Originating over four decades ago, this scale was devised by two neurosurgeons in Glasgow and continues to be extensively employed in contemporary medical practice. The GCS employs a threefold criterion-based scoring system, encompassing the assessment of the patient’s optimal eye opening (with a maximum score of 4 points), verbal response (with a maximum score of 5 points), and motor response (with a maximum score of 6 points). The summation of these individual scores yields a composite score ranging from 3 to 15, with higher values indicative of heightened levels of consciousness and neurological functionality.

The DRS is a widely recognized and validated tool for assessing functional impairment in individuals with brain damage during the course of their follow-up care. It comprises a total of eight items, each of which is evaluated on a scale ranging from 0 to either 3 or 5 points. The initial three items, namely eye opening, communication ability, and motor response, correspond to the Impairment category of the World Health Organization’s International Classification of Impairments, Disabilities, and Handicaps. The subsequent three items, namely cognitive ability pertaining to feeding, toileting, and grooming, pertain to the classification of Disability. The final two items assess psychosocial adaptability in terms of level of functioning and employability, thereby representing the category of Handicap. The summation of item ratings yields a comprehensive score ranging from 0 to 29, where higher scores indicate a more unfavorable functional outcome (e.g., 0 signifies the absence of disability, while 29 signifies an extremely vegetative state).

#### 2.5.2 EEG

The Nicolet Monitor (Nicolet, United States) was employed to gather EEG and analyze the EEG data. The EEG assessment was performed using 21 leads with disk electrodes placed according to the international 10/20 system standard. The grading criteria adhered to Hockaday et al.’s classification of disorders of consciousness (Hockaday et al., 1965). Patients in Grade I exhibited EEG waves that closely resembled normal patterns, characterized by the basic rhythm of α waves and a score of 3. Grade II patients, on the other hand, predominantly displayed θ waves with a few δ waves and a score of 2. Grade III patients exhibited predominantly δ waves with the absence of any other rhythmic activity, resulting in a score of 1. Finally, Grade IV indicated the complete absence of all waves in the patient’s EEG, warranting a score of 0.

#### 2.5.3 USEP

The EMG/NCV/EP systems (MEB-2306C, Nihon Kohden, Japan) manufactured by Nihon Kohden were employed to evaluate alterations in neurophysiology through the utilization of USEP, BAEP, and ERP (P300). The USEP evaluation involved the placement of electrodes based on the international 10/20 system, while the Greenberg criteria served as the benchmark for grading (Greenberg et al., 1977). Patients in grade I exhibited wave forms that were essentially normal, scoring a 3. Grade II patients displayed missing waveform components after 50 ms, reduced amplitude, and prolonged latency, resulting in a score of 2. Grade III patients only exhibited P15 and N20 waves, with missing waveform components after 20 ms, scoring a 1. In grade IV, either no waves were present or only P15 waves were observed, resulting in a score of 0. The USEP records nerve conduction pathways from peripheral nerves to the cortex, serving as a reliable method for evaluating a patient’s brain function in cases of DOCs.

#### 2.5.4 BAEP

The BAEP assessment was conducted using Nihon Kohden’s EMG/NCV/EP systems (MEB-2306C, Nihon Kohden, Japan), and the evaluation criteria were derived from the Greenberg criteria (Greenberg et al., 1977). The waveforms of patients classified as grade I exhibited a predominantly normal pattern, achieving a score of 3. In contrast, grade II patients displayed clear and distinguishable I-V waves; however, there was a notable prolongation in latency and a decrease in amplitude, resulting in a score of 2. Grade III patients demonstrated normal latency and amplitude for the I wave, while some of the remaining waves either appeared or exhibited indistinct positive phase waves, warranting a score of 1. Grade IV patients presented waveforms that were challenging to differentiate, or only the I wave was observable, meriting a score of 0.

#### 2.5.5 P300 event-related potentials

The P300, an event-related potentials waveform signal that is commonly studied in brain function research, is recognized as a later-stage ERP signal. It is distinguished by its latency, typically occurring approximately 300 ms after stimulus onset, and its amplitude, which typically ranges from 10 to 20 μV. Patients with dementia, psychiatric disorders, alcohol dependence, TBI, and neurodevelopmental disorders have been found to exhibit more pronounced decreases in P300 amplitude and increases in latency compared to those observed in normal aging (Davis et al., 2017; Olichney et al., 2022).

### 2.6 Statistical analysis

Continuous variables were presented as mean ± standard deviation or median [interquartile range] (IQR), while categorical variables were presented as counts and/or frequencies. The normality of continuous variables was assessed using the Shapiro–Wilk test. Comparisons were conducted using the student’s t test for normally distributed variables, and the Mann–Whitney *U*-test for variables that did not follow a normal distribution. Categorical variables were assessed using appropriate statistical tests based on sample size and minimum threshold values. The chi-square test, Pearson’s chi-square test, and Fisher’s exact test were employed for different scenarios. Furthermore, a multifactor ANOVA with interactions was utilized to examine the impact of various variables on treatment outcome indicators. The significance level was set at α = 0.05, with *p* < 0.05 indicating statistical significance. All statistical analyses were performed using SPSS 26.0 software.

## 3 Results

**Figure 3:**
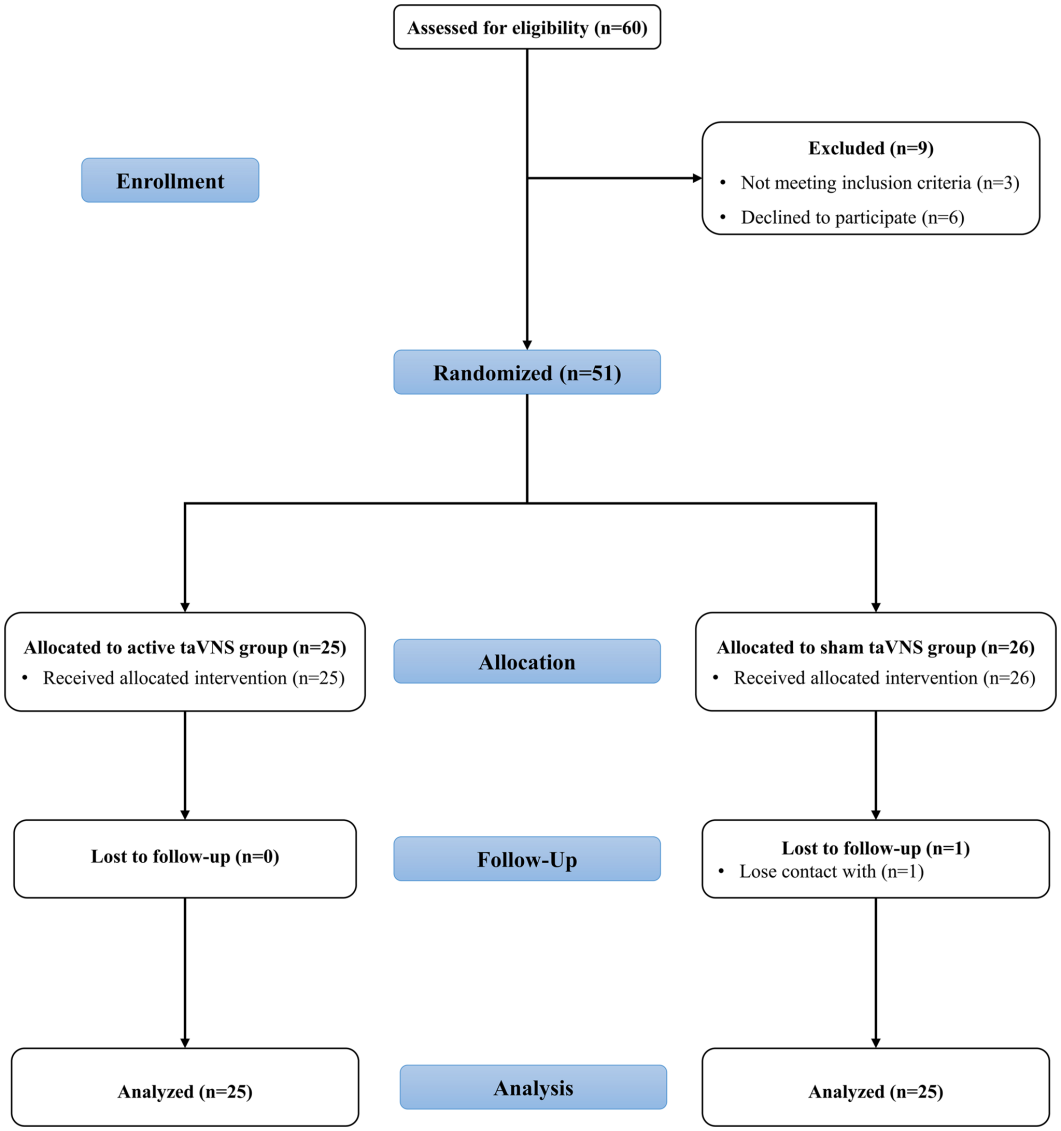
The CONSORT flowchart of the study. taVNS, transcutaneous auricular vagus nerve stimulation.

As depicted in Figure 3, the study included a cohort of 60 patients, with 9 individuals being excluded due to either not meeting the inclusion criteria (3 patients) or being unwilling or unable to participate (6 patients). Consequently, a total of 51 patients were randomly assigned to either the sham taVNS group (*n* = 25) or the active taVNS group (*n* = 26). It is important to note that throughout the study, neither the participants nor the researchers were able to discern whether the individual received taVNS or sham taVNS. During the designated follow-up period, it was observed that solely one patient from the active taVNS group became untraceable by healthcare facilities. Ultimately, a total of 25 patients from the active taVNS group and 25 patients from the sham taVNS group were included in the analysis of both behavioral and neuroelectrophysiological outcomes.

### 3.1 Baseline characteristics

The clinical demographic characteristics of patients with MCS are presented in Table 2. The age of the active taVNS group was 56 (45.5, 61) years and sham taVNS group was 55 (45.5, 61) years as indicated by the IQR. The active taVNS group comprised 10 females and 15 males, including 10 patients with TBI and 15 patients with non-TBI (13 patients with stroke, and 2 patients with HIE). In contrast, the sham taVNS group consisted of 7 females and 18 males, including 12 patients with TBI and 13 patients with non-TBI (11 patients with stroke and 2 patients with HIE). The CRS-R and GCS distribution does not conform to the normal distribution and is represented by IQR in the present study. The initial CRS-R scores were found to be 10 (9, 11) in the active taVNS group and 9 (8, 11) in the sham taVNS group. Similarly, the baseline GCS scores were 9 (9, 9) in the active taVNS group and 9 (7.5, 9) in the sham taVNS group. Additionally, the time from injury to randomization was determined to be 48 (29.5, 86.5) days in the active taVNS group and 34 (26.5, 57) days in the sham taVNS group as indicated by the IQR. There were no significant differences observed in terms of age, sex, marital status, etiology, educational attainment, willingness to undergo surgery, CRS-R and GCS scores, and time from injury to randomization between the active taVNS group and the sham taVNS group (*p* > 0.05).

**Table 2:** Baseline demographic and clinical characteristics of the participants.

Variables	Active taVNS (*N* = 25)	Sham taVNS (*N* = 25)	Statistics	
			X^2^/Z	*p*
Age, year, Median [IQR]	56 (45.5, 61)	55 (45.5, 61)	−0.049	0.961
Sex, no. (%)			0.802	0.370
Female	10 (40.00)	7 (28.00)		
Male	15 (60.00)	18 (72.00)		
Married, no. (%)			0.439	0.508
Yes	18 (72.00)	20 (80.00)		
No	7 (28.00)	5 (20.00)		
Etiology, no. (%)			0.325	0.569
Traumatic brain injury	10 (40.00)	12 (48.00)		
Non-traumatic brain injury	15 (60.00)	13 (52.00)		
Education level, no. (%)			1.292	0.524
Illiteracy or primary school	10 (40.00)	10 (40.00)		
Middle school	8 (32.00)	11 (44.00)		
High school or above	7 (28.00)	4 (16.00)		
Operation, no. (%)			0.117	0.733
Yes	20 (80.00)	19 (76.00)		
No	5 (20.00)	6 (24.00)		
Score on CRS-R, Median [IQR]	10 (9, 11)	9 (8, 11)	−1.257	0.209
Score on GCS, Median [IQR]	9 (9, 9)	9 (7.5, 9)	−1.281	0.200
Time for injury to randomization, days, Median [IQR]	48 (29.5, 86.5)	34 (26.5, 57)	−1.603	0.109

CRS-R, Coma Recovery Scale-Revised; GCS, Glasgow Coma Scale; IQR, Interquartile range; taVNS, transcutaneous auricular vagus nerve stimulation.

### 3.2 Behaviors evaluation: CRS-R, GCS, and DRS

**Figure 4:**
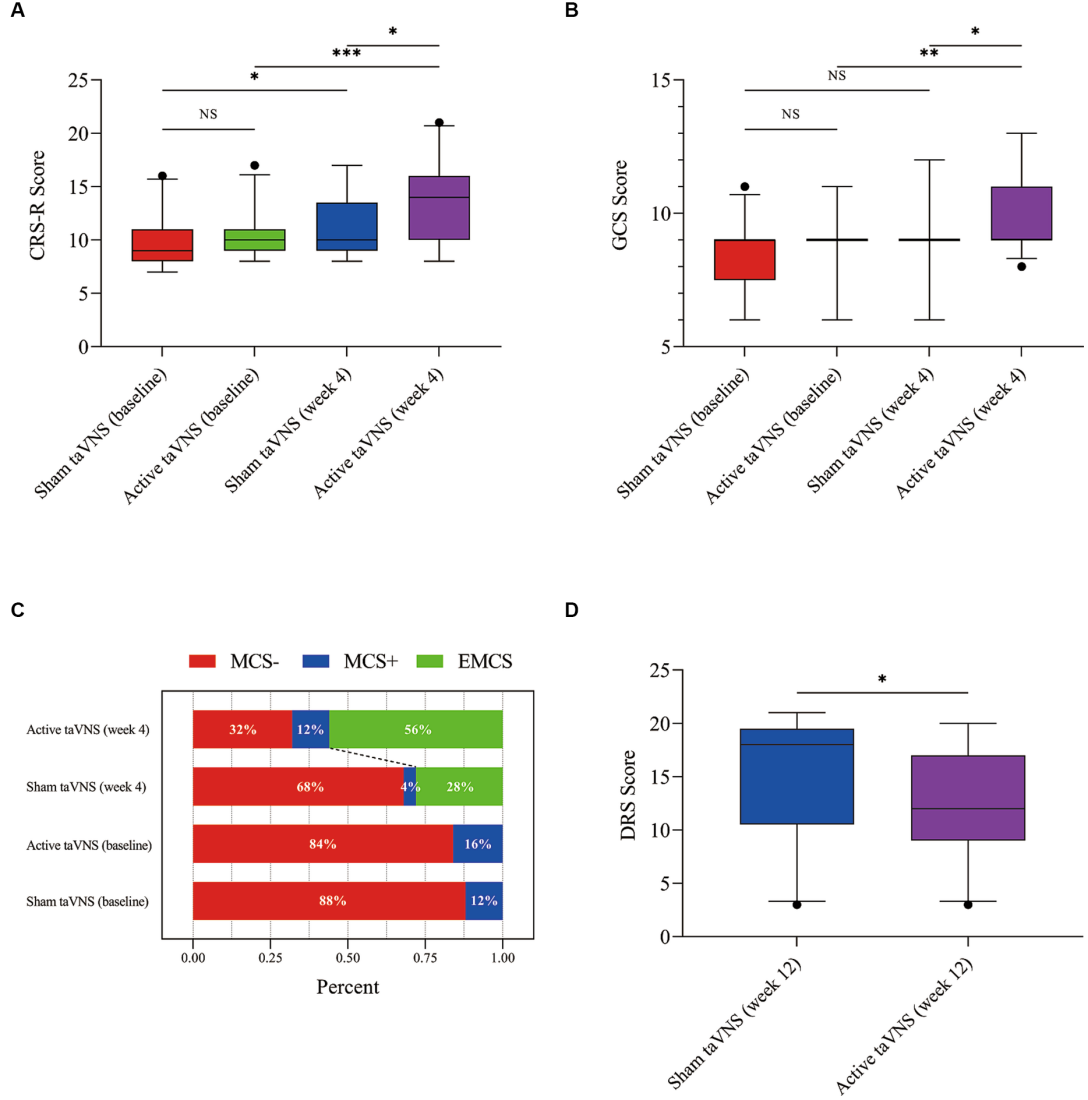
TaVNS promote consciousness recovery in patients with MCS by behavioral scales. **(A)** CRS-R. **(B)** GCS. **(C)** Proportion of consciousness level. **(D)** DRS (^*^p < 0.05, ^**^p < 0.01, ^***^p < 0.001). taVNS, transcutaneous auricular vagus nerve stimulation; MCS, minimally conscious state; CRS-R, Coma Recovery Scale-Revised; GCS, Glasgow Coma Scale; DRS, Disability Rating Scale.

According to Figure 4 and Table 3, the CRS-R scores of both the active taVNS group and the sham taVNS group exhibited significant improvement after 4 weeks of treatment compared to baseline [active taVNS group, 10 (9, 11) vs. 14 (10, 16), Z = −3.669, *p* < 0.001; sham taVNS group, 9 (8, 11) vs. 10 (9, Z = −2.810, *p* = 0.005)]. However, the active taVNS group demonstrated significantly greater recovery and achieved higher scores than the sham taVNS group [14 (10, 16) vs. 10 (9, 13.5), Z = −2.267, *p* = 0.023]. Conversely, the evaluation of GCS scores yielded slightly different results compared to the CRS-R assessment. Following 4 weeks of intervention, the active taVNS group displayed a significant improvement in GCS scores compared to baseline [9 (9, 9) vs. 9 (9, 11), Z = −2.850, *p* = 0.004], whereas no significant changes were observed in the sham taVNS group between baseline and after 4 weeks of treatment [9 (7.5, 9) vs. 9 (9, 9), Z = −1.868, *p* = 0.062]. After 4 weeks of treatment, it was observed that the active taVNS group exhibited a higher GCS score compared to the sham taVNS group [9 (9, 11) vs. 9 (9, 9), Z = −1.990, *p* = 0.047]. The state of consciousness occupancy of patients in both the active taVNS and sham taVNS groups is depicted in Figure 4C. There was no significant disparity in the percentage of conscious state between the two groups at baseline. However, following 4 weeks of treatment, a notable improvement in the state of consciousness was observed in 56% of patients in the stimulation group, which was significantly higher than the 28% improvement rate in the sham taVNS group. Moreover, the active taVNS group exhibited a significantly lower score on the DRS compared to the sham taVNS group at the 12-week follow-up [12 (9, 17) vs. 18 (10.5, 19.5), Z = −2.105, *p* = 0.035]. This finding suggests that patients with MCS in the active taVNS group demonstrate a more favorable prognosis.

**Table 3:** Between-group comparisons for behaviors evaluation of the taVNS and sham taVNS groups at all follow-ups.

Behaviors evaluation	Active taVNS (*N* = 25)	Sham taVNS (*N* = 25)	Statistics	
			Z	*P*
CRS-R, Median [IQR]				
Baseline	10 (9, 11)	9 (8, 11)	−1.257	0.209
1-week	10 (9, 12)	9 (8, 11)	−1.248	0.212
2-week	10 (9, 12.5)	9 (8, 11.5)	−1.090	0.276
3-week	13 (10, 14)	10 (8, 12.5)	−2.017	0.044
4-week	14 (10, 16)	10 (9, 13.5)	−2.267	0.023
GCS, Median [IQR]				
Baseline	9 (9, 9)	9 (7.5, 9)	−1.281	0.200
1-week	9 (9, 9)	9 (8, 9)	−1.325	0.185
2-week	9 (9, 10)	9 (9, 9)	−1.245	0.213
3-week	9 (9, 11)	9 (9, 9)	−1.848	0.065
4-week	9 (9, 11)	9 (9, 9)	−1.990	0.047
DRS, Median [IQR]				
12-week	12 (9, 17)	18 (10.5, 19.5)	−2.105	0.035

CRS-R, Coma Recovery Scale-Revised; GCS, Glasgow Coma Scale; taVNS, transcutaneous auricular vagus nerve stimulation. DRS, Disability Rating Scale; IQR, Interquartile range.

### 3.3 Subgroup analysis

**Figure 5:**
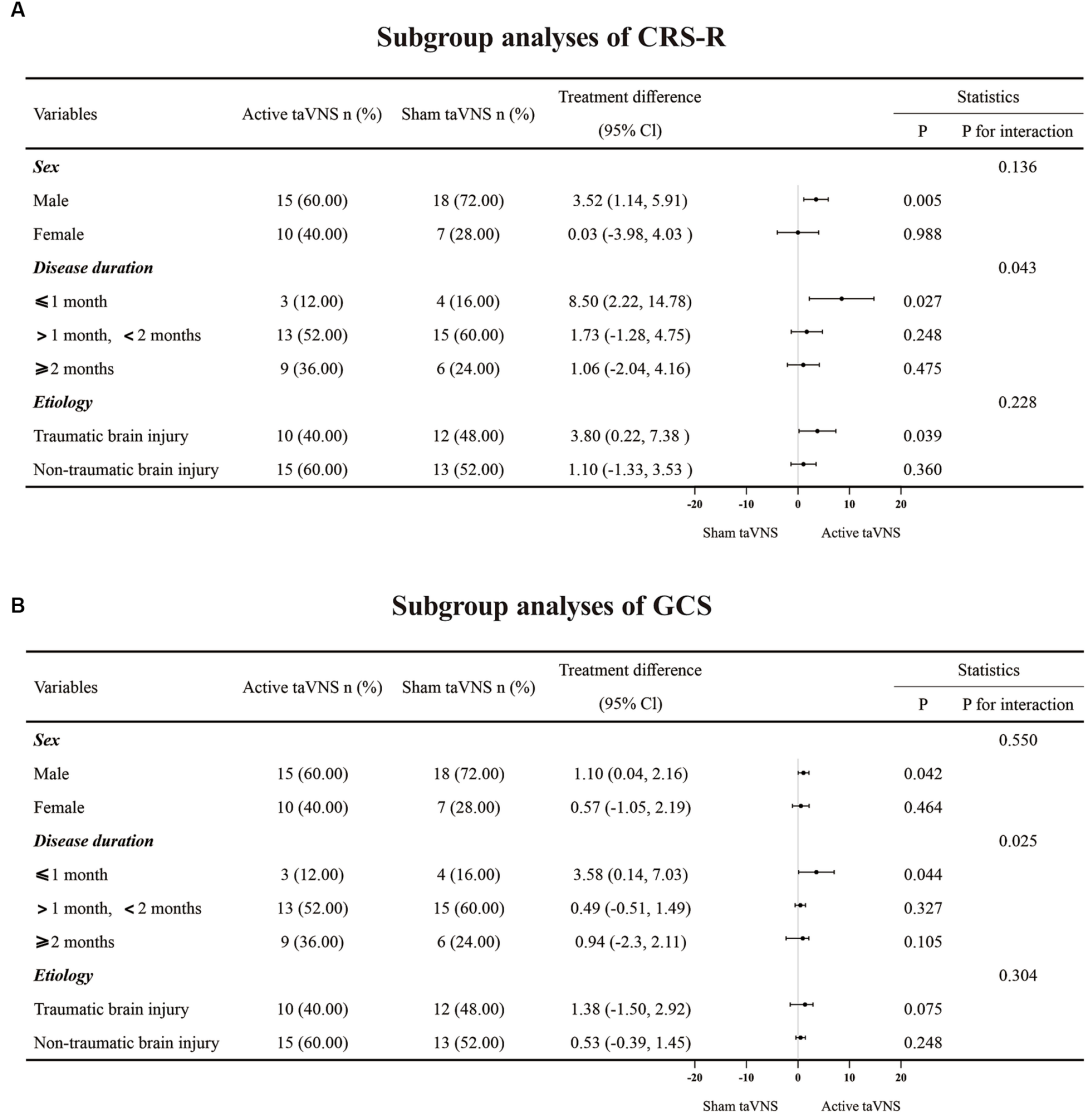
The subgroup analyses result of CRS-R and GCS. **(A)** CRS-R. **(B)** GCS. taVNS, transcutaneous auricular vagus nerve stimulation; CRS-R, Coma Recovery Scale-Revised; GCS, Glasgow Coma Scale.

In order to further investigate the factors influencing the effectiveness of taVNS treatment for patients with MCS, subgroup analysis was conducted for CRS-R and GCS (Figure 5). The analysis revealed significant interactions between disease duration and both CRS-R (P for interaction = 0.043) and GCS scores (P for interaction = 0.025), indicating that the duration of the disease has a greater impact on treatment outcomes compared to sex (CRS-R, P for interaction = 0.136; GCS, P for interaction = 0.550) and etiology (CRS-R, P for interaction = 0.228; GCS, P for interaction = 0.304). These findings suggest that MCS patients may benefit more from receiving taVNS treatment at an earlier stage.

### 3.4 Neuroelectrophysiological examination

**Figure 6:**
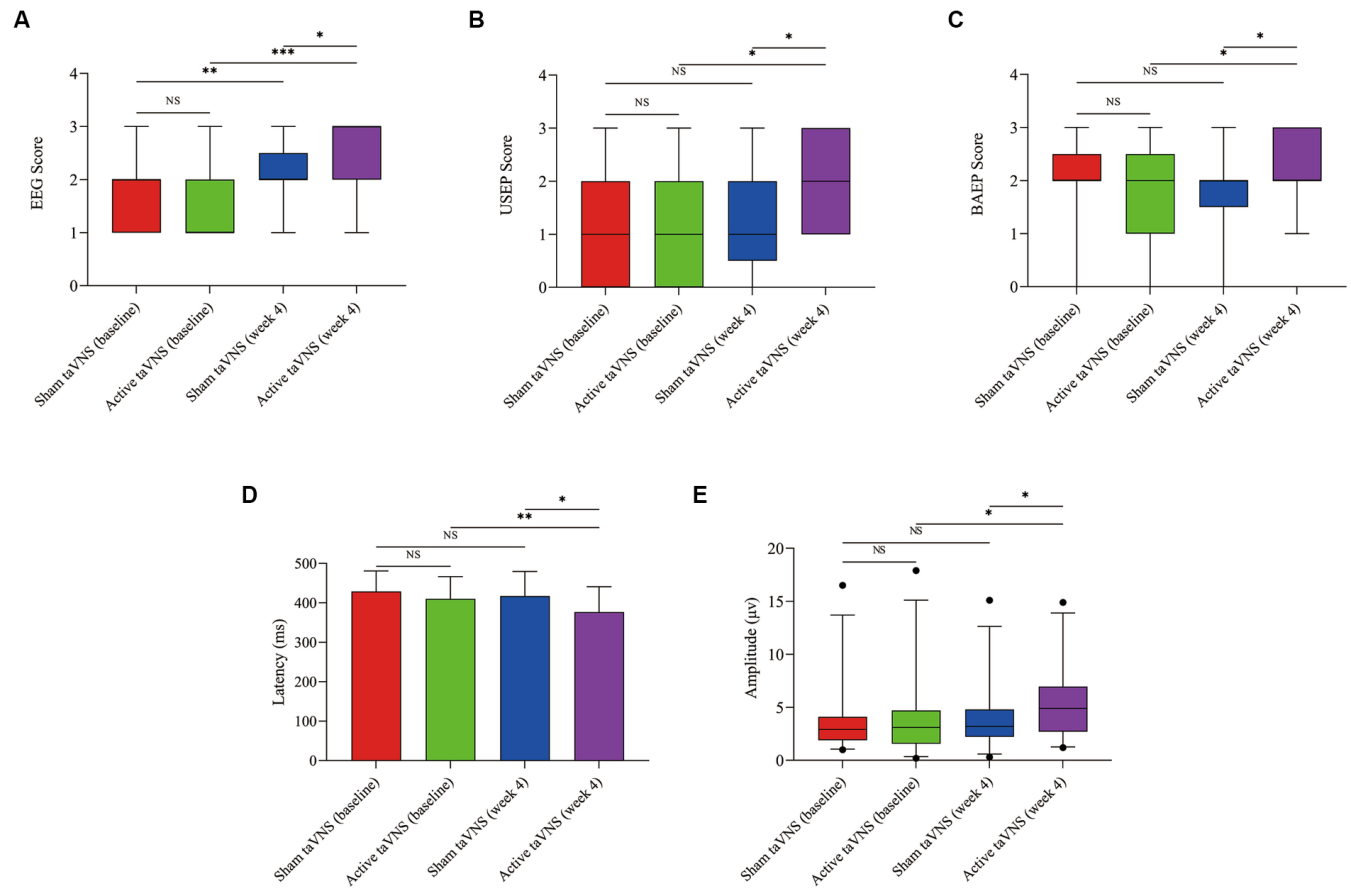
Effects of taVNS on neuroelectrophysiology in patients with MCS. **(A)** EEG. **(B)** USEP. **(C)** BAEP. **(D)** Latency of P300. **(E)** Amplitude of P300 (^*^p < 0.05, ^**^p < 0.01, ^***^p < 0.001). taVNS, transcutaneous auricular vagus nerve stimulation; EEG, electroencephalogram; USEP, upper-limb somatosensory evoked potentials; BAEP, brainstem auditory evoked potentials; P300, P300 event-related potentials.

According to the findings presented in Figure 6, the active taVNS group exhibited a significant improvement in both EEG, USEP and BAEP scores after a 4-week treatment period (EEG, Z = −3.704, *p* < 0.001; USEP, Z = −2.064, *p* = 0.039; BAEP, Z = −2.101, *p* = 0.036), surpassing the scores observed in the sham taVNS group (EEG, Z = −2.086, *p* = 0.037; USEP, Z = −2.014, *p* = 0.044; BEAP, Z = −2.298, *p* = 0.022). Additionally, the sham taVNS group also showed the improvement in EEG at week 4 compare with baseline (EEG, Z = −2.828, *p* = 0.005). Conversely, the USEP and BAEP scores in the sham taVNS group did not display any significant differences before and after treatment (USEP, Z = −0.843, *p* = 0.457; BAEP, Z = −1.032, *p* = 0.302). In order to assess the enhancement of consciousness levels, we utilized the shortened latency and increased wave amplitude of the P300. After a duration of 4 weeks of treatment, it was observed that the active taVNS group exhibited a significantly reduced P300 latency compared to their baseline measurements (t = 2.872, *p* = 0.008), as well as a lower latency compared to the sham taVNS group (t = 2.275, *p* = 0.027). Additionally, the active taVNS group displayed a significantly increased P300 wave amplitude from baseline (Z = -1.992, *p* = 0.046), surpassing the amplitude observed in the sham taVNS group (Z = −1.974, *p* = 0.049). Conversely, the sham taVNS group exhibited a shorter P300 latency and an elevated amplitude from baseline, although these differences were not found to be statistically significant (P300 latency, t = 0.767, *p* = 0.451; P300 amplitude, Z = −0.543, *p* = 0.587). These findings suggest that taVNS may have an impact on brain activity, potentially leading to improvements in levels of consciousness.

### 3.5 Side effects

Some side effects such as pneumonitis and constipation occurred while the study was in progress. However, no significant differences were observed in the occurrence of these symptoms among the active taVNS group and the sham taVNS group in relation to the central nervous system, respiratory system, urinary system, gastrointestinal system, cardiovascular system, and blood system, as indicated in Table 4. These findings suggest that taVNS may be considered a safe method, as no significant adverse events related to taVNS were observed during treatment.

**Table 4:** Adverse events in two groups.

Adverse events	Active taVNS (*N* = 25)	Sham taVNS (*N* = 25)	Statistics	
			X^2^	*P*
Central nervous system (%)			2.304	1.000
Seizure	2 (8.00)	1 (4.00)		
PSH	0 (0.00)	1 (4.00)		
Hydrocephalus	1 (4.00)	0 (0.00)		
Encephalitis infection	0 (0.00)	0 (0.00)		
Respiratory system (%)			0.802	0.370
Pneumonitis	15 (60.00)	18 (72.00)		
Pneumothorax	0 (0.00)	0 (0.00)		
Urinary system (%)				1.000
Bladder infection	1 (4.00)	2 (8.00)		
Acute kidney injury	0 (0.00)	0 (0.00)		
Gastrointestinal system (%)			2.154	0.588
Gastric hemorrhage	1 (4.00)	0 (0.00)		
Alanine aminotransferase increased	1 (4.00)	2 (8.00)		
Constipation	8 (32.00)	11 (44.00)		
Cardiovascular system (%)			2.665	0.801
Deep vein thrombosis	2 (8.00)	1 (4.00)		
Pulmonary embolism	0 (0.00)	1 (4.00)		
Abnormal blood pressure	5 (20.00)	7 (28.00)		
Sinus bradycardia	1 (4.00)	0 (0.00)		
Blood system (%)			1.870	1.000
Anemia	1 (4.00)	0 (0.00)		
Coagulant abnormalities	0 (0.00)	1 (4.00)		
Bloodstream infection	0 (0.00)	0 (0.00)		
Others (%)			0.948	0.711
Ear skin ulceration	0 (0.00)	0 (0.00)		
Electrolyte disturbance	2 (8.00)	1 (4.00)		
Hypoalbuminemia	3 (12.00)	5 (20.00)		

PSH, paroxysmal sympathetic hyperactivity; taVNS, transcutaneous auricular vagus nerve stimulation.

## 4 Discussion

In the current investigation, it was observed that the active taVNS group exhibited notable enhancements in both total CRS-R and GCS scores at the fourth week in comparison to the sham taVNS group. Furthermore, during the twelfth week follow-up, the active taVNS group displayed lower scores on the DRS in contrast to the sham group, indicating a significant improvement in functional recovery due to taVNS treatment as opposed to sham treatment. Moreover, the neuroelectrophysiological assessment encompassing EEG, USEP, BAEP, and P300 demonstrated a notable improvement in the active taVNS group at the fourth week when compared to the sham group. The data presented in this study offer evidence suggesting that taVNS has the potential to enhance consciousness and facilitate functional rehabilitation in individuals diagnosed with MCS, through its modulation of brain activity and activation of neural conduction pathways.

The findings from the CRS-R score indicate that the patients in the active taVNS group exhibited a more favorable recovery of consciousness compared to those in the sham group after a duration of 3 weeks of treatment. Additionally, the GCS score in the taVNS group demonstrated a divergence from the sham group after 4 weeks of treatment. Furthermore, it is noteworthy that the overall CRS-R score displayed a substantial improvement in the sham taVNS group following 4 weeks of treatment in comparison to the baseline, while no significant difference was observed in the GCS score before and after treatment. The disparity in outcomes may be attributed to the CRS-R’s superior sensitivity compared to the GCS in assessing consciousness following brain injury, as well as the enhanced construct validity of the hierarchical structure of its six subscales (Bodien et al., 2016; Zhang et al., 2019). The temporal and recovery patterns of this therapeutic impact, as measured by CRS-R, GCS, and DRS scores, align with prior investigations on median nerve stimulation (Lei et al., 2015; Wu et al., 2023) and trigeminal nerve stimulation (Ma et al., 2023). Nevertheless, variations exist in the timing of injury occurrence and the duration of intervention across these clinical studies. For instance, Wu et al. exclusively incorporated individuals with acute coma who had experienced TBI within a period of 7–14 days in their clinical investigation involving median nerve stimulation, which was administered for 8 h daily over a span of 2 weeks (Wu et al., 2023). In contrast, our study entailed a 4-week intervention period of 60 min per day, and the patients included had surpassed 28 days post-injury. Given that the recovery duration for patients with DOCs typically falls within the range of 1.5 months to 1 year following the injury (Giacino et al., 2020; McCrea et al., 2021), a more extensive observation and intervention period in our study lends greater credibility to elucidating the therapeutic impact. Despite the lack of consistency in the stimulation parameters of taVNS in current clinical studies, the determination of optimal parameters for taVNS in the treatment of patients with DOCs necessitates further research. Furthermore, it has been observed that patients with MCS who receive taVNS treatment within a month exhibit superior effects compared to those treated for longer durations in the present study, suggesting that early taVNS intervention may lead to heightened levels of consciousness and improved functional recovery in MCS patients.

There exists a considerable body of research that examines the application of taVNS as a means to facilitate the restoration of consciousness in patients diagnosed with DOCs (Dong et al., 2023). However, the current literature lacks a sufficient number of high-quality randomized controlled clinical trials in this area. Yu et al. (2017) published a case report detailing the progress of a 73-year-old female patient who suffered from respiratory and cardiac arrests. Notably, following a 4-week taVNS intervention, the patient’s level of consciousness improved from 6 points (VS/UWS) to 13 points (MCS) on the CRS-R. Hakon et al. (2020) conducted a study involving 3 patients in a VS/UWS and 2 patients in a MCS who had previously experienced diffuse axonal injury more than 28 days prior. After an 8-week taVNS intervention, 3 patients exhibited improvement in the CRS-R, with 2 MCS patients transitioning to a higher level of consciousness and 1 VS/UWS patient progressing to MCS. Additionally, Noe et al. (2020) conducted a separate study to investigate the feasibility, safety, and therapeutic effects of taVNS treatment in patients with DOCs following brain injury. During the four-week period preceding taVNS treatment, no discernible changes were observed in the CRS-R scores of the patients. However, upon completion of the one-month follow-up, a notable and statistically significant increase in CRS-R scores was observed. It is worth mentioning that none of the patients diagnosed with VS/UWS demonstrated any alterations in their CRS-R scores, whereas a progressive elevation in CRS-R scores was observed in five out of the eight patients diagnosed with MCS throughout the duration of this study. Yu et al. (2021) undertook an initial investigation to explore the cerebral hemodynamic associations of taVNS in the reinstatement of consciousness. Those patients who displayed a reaction to auditory stimuli exhibited a positive result on the GCS after undergoing a four-week taVNS intervention. Conversely, patients who did not exhibit a response to auditory stimuli encountered unfavorable outcomes. Osinska et al. (2022) conducted a case study in which they observed a patient who experienced a restoration of impaired consciousness after undergoing 6 months of taVNS treatment. The individual in question was a 28-year-old female who had previously been diagnosed with VS/UWS based on a four-point assessment using the CRS-R following a TBI that had occurred 6 years prior. It is worth noting that the patient’s CRS-R score exhibited a significant improvement from 4 to 13 points after approximately 100 days of taVNS therapy, indicating a potential transition from VS/UWS to MCS. Yifei et al. (2022) conducted a study examining the impact of taVNS on a cohort of 12 patients with DOCs resulting from acquired brain injury. Over a period of 14 days, taVNS was administered to the patients, yet no significant improvements were observed on the CRS-R. However, analysis of the resting state EEG power spectrum revealed a decrease in delta band energy and an increase in beta band energy among patients diagnosed with MCS, in contrast to those diagnosed with VS/UWS.

Furthermore, a clinical trial was conducted in our previous study to examine the therapeutic effectiveness of taVNS in individuals diagnosed with DOCs (Zhou et al., 2023). The study encompassed a cohort of 57 patients with DOCs, consisting of 25 patients in a VS/UWS and 32 patients in a MCS, all of whom had experienced acquired brain injuries. The results of this study provide evidence indicating that taVNS may have the potential to be an effective method for facilitating the restoration of consciousness in patients diagnosed with MCS compare with VS/UWS. The reason for this may be that the duration period of taVNS operation is too short. In order to further investigate the effects of taVNS treatment in MCS patients, we designed the present study. Our findings demonstrate that patients in the active taVNS group exhibited higher scores on the CRS-R and GCS compared to the sham taVNS group after 4 weeks of intervention. Additionally, they also demonstrated better outcomes at the 12-week follow-up assessment using the DRS. The results of this study suggest that taVNS could promote consciousness recovery and may improve the prognosis of patients with MCS.

Recent studies have emphasized the potential impact of taVNS on the process of consciousness restoration in patients with DOCs. However, the precise mechanism by which taVNS contributes to the recovery of consciousness remains unclear. As an innovative and non-invasive neuromodulation technique, taVNS elicits therapeutic effects by stimulating the afferent branches of the vagus nerve located in the skin of the ear. Building upon the concepts of consciousness recovery and taVNS mechanisms, Briand et al. (2020) have proposed the Vagus Cortical Pathways model. The authors posited several potential mechanisms that could elucidate the therapeutic effects of taVNS on brain activity during the process of consciousness recovery. These mechanisms include the activation of the Ascending Reticular Activating System (ARAS), the activation of the thalamus, the re-establishment of the cortico-striatal-thalamic-cortical loop, the promotion of negative connectivity between the external and default mode networks through the activation of the salience network, and the increase in activity and connectivity within the external network via the norepinephrine (NE), in addition, serotonin pathways are more active within the default mode network. In addition, taVNS could be an enhancer of both cortical arousal.

and alertness by modulating EEG alpha oscillations (Chen et al., 2023). In the present study, it was observed that the active taVNS group exhibited significant enhancements in both USEP and BAEP compared to the sham group. These findings of present study provide evidence suggesting that taVNS has the potential to stimulate the pathways connecting peripheral nerves to the cortex, as well as the ARAS pathway.

Other potential mechanisms also exist. Firstly, numerous studies have suggested that vagus nerve stimulation may elicit alterations in neurotransmitters, such as NE (Hulsey et al., 2019; Berger et al., 2021) and orexin-A (Dong and Feng, 2018), leading to modifications in brain activity’s electrical signals. NE and orexin-A are excitatory neurotransmitters involved in the regulation of sleep-wakefulness. Prior research has demonstrated that augmenting the concentration of NE and orexin-A in the brain can enhance functional recovery and elevate the level of consciousness (Clauss, 2014; Tang et al., 2019). Prior research has demonstrated that augmenting the levels of NE and orexin-A in the brain can facilitate functional recuperation and enhance the state of awareness (Jia et al., 2012; Fukabori et al., 2020). Electrophysiological markers in the form of P300 can provide corroborative evidence supporting the notion that taVNS may augment the release of NE in the central nervous system (Schutte et al., 2020). Our findings indicate that taVNS has the potential to ameliorate the amplitude and reduce the latency of P300, suggesting that taVNS might influence NE release to promote the recovery of consciousness. Furthermore, our previous investigation provided evidence that VNS has the potential to awaken comatose rats with TBI through the upregulation of orexin-A expression in the prefrontal cortex (Dong and Feng, 2018). Moreover, it is plausible that the effects of taVNS involve an increase in cerebral blood flow (CBF). This notion is supported by the findings of Peng et al. (2018), who observed immediate and significant blood oxygenation level dependent (BOLD) signal changes in the prefrontal, auditory, and limbic cortices of healthy individuals following taVNS, as detected by functional magnetic resonance imaging (fMRI). Furthermore, taVNS exhibited a notable enhancement in CBF within various brain regions, including the thalamus, superior temporal gyrus, and hippocampus, among patients with DOCs (Yu et al., 2021). Additionally, a large number of studies have showed that taVNS could inhibit central and peripheral inflammation, which is possibly related to the effectiveness of taVNS in reducing brain tissue damage (Zhao et al., 2012; Wang et al., 2021; Go et al., 2022). Therefore, it is reasonable to hypothesize that the application of taVNS may alleviate the adverse effects of TBI by attenuating inflammation, oxidative stress, and apoptosis (Liu et al., 2020; Tang et al., 2020; Wang et al., 2021b).

However, our study has several limitations that should be acknowledged. Firstly, the duration of the taVNS treatment period and the follow-up time were relatively short, which hinders the ability to ascertain the long-term effects of taVNS on patients with MCS. It is crucial to observe the long-term effects of taVNS treatment in both MCS and VS/UWS patients. Secondly, the secondary outcome we used in this study is only neuroelectrophysiology, neuroimaging assessment and serum biomarkers were lacking such as fMRI and interleukin. Lastly, future research should consider incorporating a larger sample size and conducting multicenter studies to improve the generalizability and reliability of the findings.

## 5 Conclusion

In summary, this clinical trial has yielded evidence suggesting that taVNS is a potentially effective and safe intervention for individuals diagnosed with MCS. The observed improvements in brain activity and neural conduction pathways indicate that taVNS may contribute to the restoration of consciousness. Consequently, these findings present a promising therapeutic approach for patients afflicted with MCS.

## Data availability statement

The original contributions presented in the study are included in the article/Supplementary material, further inquiries can be directed to the corresponding authors.

## Ethics statement

The studies involving humans were approved by the Ethics Committee of the First Affiliated Hospital of Nanchang University ((2022)CDYFYYLK(06–031)). The studies were conducted in accordance with the local legislation and institutional requirements. The participants provided their written informed consent to participate in this study.

## Author contributions

YZ: Data curation, Investigation, Methodology, Project administration, Writing – original draft, Writing – review & editing, Formal Analysis, Software. YS: Data curation, Formal Analysis, Methodology, Project administration, Software, Writing – original draft, Writing – review & editing, Validation. PH: Methodology, Software, Writing – original draft, Writing – review & editing, Investigation. QX: Methodology, Software, Writing – original draft, Writing – review & editing, Data curation. JK: Software, Writing – original draft, Writing – review & editing. YT: Software, Writing – original draft, Writing – review & editing, Formal Analysis, Investigation, Methodology, Supervision. ZF: Formal Analysis, Investigation, Methodology, Visualization, Writing – original draft, Writing – review & editing, Conceptualization, Project administration, Resources, Supervision, Validation. XD: Conceptualization, Formal Analysis, Investigation, Methodology, Project administration, Resources, Supervision, Validation, Visualization, Writing – original draft, Writing – review & editing, Data curation, Funding acquisition.
